# Cefepime-Induced Nonconvulsive Status Epilepticus in a Pediatric Patient with Normal Renal Function

**DOI:** 10.1177/2329048X221119575

**Published:** 2022-08-07

**Authors:** Clever Nguyen, Taylor Clegg, Ashutosh Kumar, Sita Paudel

**Affiliations:** 1 12310 Penn State College of Medicine, Hershey, PA, USA; 2 Department of Pediatrics and Neurology, Penn State Health Milton S. Hershey Medical Center, Hershey, PA, USA

**Keywords:** cefepime, neurotoxicity, nonconvulsive status epilepticus, pediatric, EEG

## Abstract

**Introduction:** Cefepime, a fourth-generation cephalosporin, is known to risk the induction of neurotoxic impairment from confusion to nonconvulsive status epilepticus (NCSE). Neurotoxic effects of cefepime are most commonly evident in the setting of impaired renal function in adults; however, are rarely present in those with normal renal excretion function or in the pediatric population. **Case:** We present a case of a 16-year-old female with a complicated past medical history but no accounts of impaired renal function yet, after starting cefepime, presented with encephalopathy, intermittent stimulus-induced posturing, and was found to have NCSE. Discontinuation of cefepime and administration of additional antiepileptics provided significant improvement in EEG and allowed the patient to return to baseline within two days. **Conclusion:** Cefepime-induced nonconvulsive status epilepticus should be considered in any patient with or without impaired renal function that shows acute changes in mental status, and/or reduced consciousness, after initiating cefepime treatment.

## Introduction

Cefepime is a fourth-generation cephalosporin commonly used in the hospital setting due to its broad antimicrobial coverage. Because of its ability to cross the blood-brain barrier, several central nervous system side effects have been reported, including nonconvulsive status epilepticus (NCSE).^1^ The majority of cases reported are in patients with impaired renal function; however, a few cases of patients with intact renal function have been published. Moreover, most reported cases occurred in adult and older populations.^1^ A few pediatric cases of cefepime-induced neurotoxicity have been reported, but only in patients with renal dysfunction.^2–5^ Here, we report the case of a 16-year-old female with a normal renal function who developed NSCE after treatment with cefepime.

## Case Report

Our patient is a 16-year-old female with a weight of 32.6 kg and a complicated medical history including chromosome 10-15 unbalanced translocation, spastic quadriplegic cerebral palsy, epilepsy, hydrocephalus with VP shunt, tethered cord syndrome; status post-surgery at age 2, chronic respiratory failure with tracheostomy and intermittent ventilator dependence, feeding intolerance with G-tube placement at age 2 weeks with fundoplication, scoliosis, and thoracic lordosis status post spinal fusion, and global developmental delay. She underwent conversion of her VP (ventriculopeitoneal) shunt to VA (ventriculoatiral) shunt in December 2021, complicated by an episode of nonsustained ventricular tachycardia after placement, and then a revision shortly after. She then presented in February 2022 with concerns for wound breakdown over the shunt valve. Inspection of the site showed skin breakdown, redness, improper drainage, and swelling. The patient was started on cefepime 1630 mg IV (intravenous) q8h (every 8 h), linezolid 320 mg IV q12h, and vancomycin 125 mg G-tube bid (twice daily) while an infectious workup was pending. Three days after admission, the patient was taken to the OR (operation room) for a left frontal conversion to a left parietal VA shunt. Linezolid was later stopped due to negative blood/CSF cultures, but cefepime and vancomycin were continued due to ongoing concerns for positive *Escherichia coli* urine cultures, positive *Pseudomonas spp.* respiratory cultures, and *Clostridium difficile* prophylaxis. Her renal function remained stable throughout her hospital course indicated by daily creatinine concentrations ranging in between 0.63–0.84 mg/dL, BUNs between 10–19 mg/dL, and an estimated pediatric GFRs of 63.45–84.60 mL/min.

**Figure 1.**
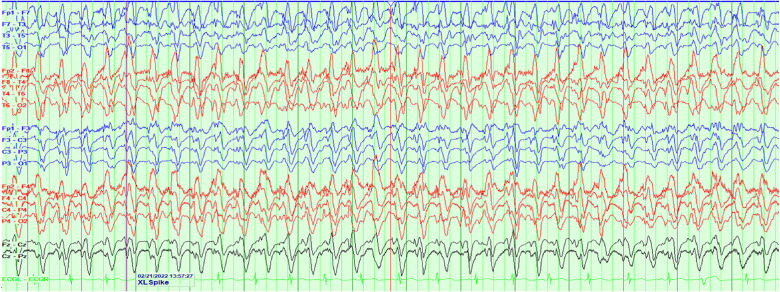
EEG demonstrating continuous generalized rhythmic 2.5-3 Hz epileptiform discharges suggestive of nonconvulsive status epilepticus three days after starting cefepime.

Post-operatively, the patient was less responsive than usual with higher blood pressures and tachycardia. Parents reported that she usually is happy and able to interact with them by smiling, looking at them, and hugging them; however, since returning from the OR, they stated it seemed as if she is “not there” and not able to recognize them. Over the next two nights, her parents noted that she had frequent twitching whenever she was touched. On physical exam, three days post-op, she was afebrile and other vitals were stable. Systemic examination was essentially unremarkable except tracheostomy and G-tube in place. On neurological examination, her eyes were open but she was not responsive to stimuli. Pupils were reactive to light but was not able to track. Eyes were midline with continuous horizontal nystagmus. On motor exam, increased tone and decreased muscle bulk were noted in all four extremities and there were no purposeful movement. Intermittent extensor posturing was noted with tactile stimuli, otherwise there was no other response to noxious stimuli. Her reflexes were brisk throughout, and toes were upgoing bilaterally. Investigative initial postsurgical CT head showed expected pneumocephalus with mild soft tissue swelling over the left frontal scalp. Additionally, a repeat CT two days later showed decreased pneumocephalus and mild soft tissue swelling with no evidence of hydrocephalus. Continuous EEG was then pursued, revealing relentless rhythmic 2.5-3 Hz generalized epileptiform discharges suggestive of nonconvulsive status epilepticus (Figure 1).

**Figure 2.**
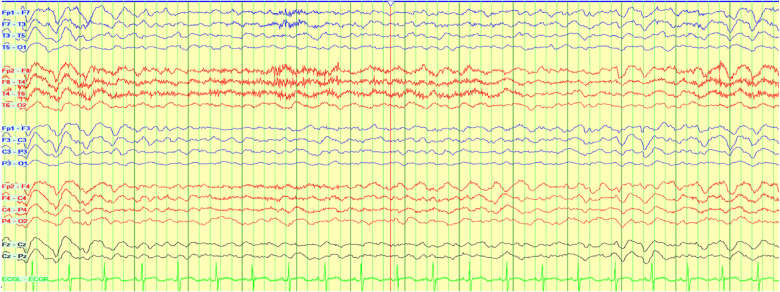
EEG illustrating electroencephalographic improvement associated with clinical improvement two days after discontinuing cefepime as the patient returns to baseline.

There was no clear evolution on the EEG with associated clinical manifestation, however, this pattern was observed >50% time of the total study. Cefepime was discontinued with the suspicion of cefepime-related neurotoxicity. To further address the immediate presentation of NCSE, the patient was additionally provided doses of levetiracetam, lorazepam, and phenobarbital. Over the next two days, the patient demonstrated dramatical improvement on EEG monitoring and on exam, appearing to be back to her baseline state of health (Figure 2).

##  Discussion

Cefepime, a fourth-generation cephalosporin, is commonly utilized in the hospital setting due to its broad antimicrobial coverage of gram-negative, gram-positive, and antipseudomonal activity.^6^ By inhibiting bacterial cell wall synthesis and inactivating penicillin-binding proteins, cefepime is effective in inducing autolysis in multiple common pathogens such as *E. coli* and *Pseudomonas spp.*; however, it is known that cefepime can also cross the blood-brain-barrier to antagonize gamma-aminobutyric acid (GABA) receptors.^7^ For this reason, cefepime is used throughout medicine to treat an array of illnesses and complications such as pneumonia, urinary tract infections, and skin infections, but in rare instances, induces unwanted neurotoxic side effects.

Cefepime is usually well tolerated by both pediatric and adult populations due to its short half-life and 85% kidney clearance when provided parenterally and when dose adjusted for patients with renal dysfunction.^8^ Standard practices of cefepime dosing of patients with normal renal function are defined as having a GFR > 60 with cefepime administration set as 1 to 2 g every 8 h, and for patients with impaired renal function, defined as GFR < 60, is 0.5 to 2 grams every 12 to 24 h.^9^ In the pediatric population of normal renal function, cefepime administration utilizes 50 mg/kg IV every 8 h or 12 h for non-CNS conditions such as pneumonia, urinary tract infections, and skin/subcutaneous infections, and for CNS conditions such as bacterial meningitis, 150 mg/kg/day IV cefepime dosing is encouraged.^10^ In pediatric patients with renal impairment, data is not readily available to make dose adjustment recommendation in relation to pediatric creatinine clearance, and current recommendations are to make dosage modifications proportional to adjustments made for adults.^10^ Instances of cefepime-induced neurotoxicity evidenced by encephalopathy or non-convulsive status epilepticus most commonly occur in clinical settings where dose adjustments are not made for patients with renal dysfunction.^9^ In addition, most reports of cefepime-induced neurotoxicity have been described in the adult population with renal impairment.^11^ However, more recent discussion has called into light the prevalence of cefepime-induced neurotoxicity occurrence within the pediatric population on hemodialysis.^12^ Our case report furthers the conversation and highlights the significance of considering and pursuing the differential of cefepime-induced neurotoxicity even in pediatric patients with intact renal clearance.

In the absence of renal impairment in our patient's hospital course, *E. coli* and *Pseudomonas* infections post-op indicated the use of cefepime in our infectious disease antibiotic recommendation. Moreover, cefepime-induced neurotoxicity should be of high suspicion in any clinical setting where new-onset altered mental status in recent administration of cefepime regardless of patient age or renal status. EEG may be insightful in providing evidence of encephalopathy via the presence of triphasic waves or NCSE evident by ≤ 2.5 Hz epileptiform discharges.^13^ Obtaining lab values for cefepime serum concentration may also be useful in exploring cefepime-induced neurotoxicity, though this lab order is not widely practiced. Excessive cefepime exposure is defined by serum trough concentrations of >20 μm/mL with a median trough concentration of 38 μm/mL upon cefepime-related neurotoxicity.^14^ Our case represents the possibility of cefepime-induced neurotoxicity leading to NCSE, however given patient's complicated medical history, other factors might be confounding. Nevertheless, treatment of suspected cefepime-induced neurotoxicity would be terminating active cefepime administration and providing benzodiazepine to abort NCSE. Due to possibility of cefepime-induced neurotoxicity, albeit it relatively rare, providers should keep into consideration for other antimicrobial agents that provides broad-spectrum coverage and a relatively lower risk for neurotoxicity in a patient specific setting.^15^

## Conclusion

The possibility of cefepime-induced nonconvulsive status epilepticus should not be overlooked in any patient with or without impaired renal function that shows acute changes in mental status, reduced consciousness, and posturing after starting cefepime treatment. In addition to following appropriate multi-organ-based workups for encephalopathy in pediatric patients, it is imperative to consider a continuous EEG to reveal possible underlying epileptiform activity. In the cases of abnormal EEG findings that support cefepime-induced NCSE or neurotoxicity, cefepime should be discontinued and benzodiazepines are indicated to resolve NCSE.
